# ASO Author Reflections: Imaging-Guided Localization Does Not Improve the Performance of Tailored Axillary Surgery—Preplanned OPBC-03/TAXIS Substudy

**DOI:** 10.1245/s10434-023-14437-9

**Published:** 2023-12-01

**Authors:** Martin Heidinger, Walter P. Weber

**Affiliations:** 1 Breast Center, University Hospital Basel, Basel, Switzerland; 2 University of Basel, Basel, Switzerland

## Past

Axillary lymph node dissection (ALND) is standard of care for patients with clinically node-positive (cN+) breast cancer (BC) in the upfront surgery setting and in case of residual nodal disease (ypN+) after neoadjuvant chemotherapy (NACT). In patients with cN+ disease and good clinical response, targeted axillary dissection (TAD) was introduced to determine pathologic complete response after NACT.^1,2^ TAD allows the selective removal of the previously sampled and clipped lymph node (LN) by use of imaging-guided localization (IGL) in combination with the sentinel lymph node (SLN) procedure. TAD significantly reduced the false-negative rate (FNR) compared with the SLN procedure alone in this setting.^1,3^ In the international, multicenter phase-III OPBC-03/TAXIS trial including cN+ patients in the upfront surgery setting and in case of residual disease after NACT, patients are randomized to undergo tailored axillary surgery (TAS) in combination with either axillary radiotherapy (ART), or immediate back-up ALND.^4^ TAS consists of the removal of the SLN as well as all palpably suspicious nodes, while the use of IGL is optional. The present pre-specified substudy investigated the impact of IGL on the performance of TAS.

## Present

We included 500 patients from 44 study sites between August 2018 and June 2022. Of those, 67.0% (335/500) underwent upfront surgery, 30.2% (151/500) had ypN+ disease after NACT and 2.8% (16/500) underwent neoadjuvant treatment other than chemotherapy. IGL was attempted in 84.4% (422/500) of patients and successfully performed in 97.9% (413/422). No difference between TAS with or without IGL was found in the total number of removed nodes (5 [IQR 3–8] vs 4 [IQR 3–6]; *p* = 0.3) and positive nodes (2 [IQR 1–4] vs 2 [IQR 1–3]; *p* = 0.6). The proportion of patients with residual nodal disease removed by ALND after TAS did not differ by use of IGL (with IGL 68.0% vs without IGL 57.6%, *p* = 0.2).^5^

## Future

Even though IGL was technically successful in almost all patients, it did not significantly improve the performance of TAS. Therefore, IGL will not become a mandatory element of TAS. The clinical relevance of reducing the false-negative rate by IGL in the diagnostic setting of complete nodal response remains unclear.

## References

[1] Caudle AS, Yang WT, Krishnamurthy S (2016). Improved axillary evaluation following neoadjuvant therapy for patients with node-positive breast cancer using selective evaluation of clipped nodes: implementation of targeted axillary dissection. J Clin Oncol.

[2] Kuemmel S, Heil J, Bruzas S (2023). Safety of targeted axillary dissection after neoadjuvant therapy in patients with node-positive breast cancer. JAMA Surg.

[3] van der Noordaa MEM, van Duijnhoven FH, Straver ME (2018). Major reduction in axillary lymph node dissections after neoadjuvant systemic therapy for node-positive breast cancer by combining PET/CT and the MARI procedure. Ann Surg Oncol.

[4] Henke G, Knauer M, Ribi K (2018). Tailored axillary surgery with or without axillary lymph node dissection followed by radiotherapy in patients with clinically node-positive breast cancer (TAXIS): study protocol for a multicenter, randomized phase-III trial. Trials..

[5] Weber W, Heidinger M, Hayoz S (2023). Impact of imaging-guided localization on performance of tailored axillary surgery in patients with clinically node-positive breast cancer: prospective cohort study within TAXIS (OPBC-03, SAKK 23/16, IBCSG 57–18, ABCSG-53, GBG 101). Ann Surg Oncol.

